# Nanobodies: a tool to open new horizons in diagnosis and treatment of prostate cancer

**DOI:** 10.1186/s12935-021-02285-0

**Published:** 2021-10-30

**Authors:** Maryam Hosseindokht, Hamid Bakherad, Hamed Zare

**Affiliations:** 1 Cellular and Molecular Research Center, Birjand University of Medical Sciences, Birjand, Iran; 2 Department of Pharmaceutical Biotechnology and Isfahan Pharmaceutical Sciences Research Center, School of Pharmacy and Pharmaceutical Sciences, Isfahan University of Medical Sciences, Isfahan, Iran

**Keywords:** Nanobody, VHH, Prostate cancer, PSMA, PSA

## Abstract

**Background:**

Prostate cancer is one of the most common cancers in men and its incidence has increased dramatically in the last decade. This increase in the detection of this type of cancer is based more on the detection of PSA or PSMA antigens as the most important specific antigens of this cancer, and this early detection has greatly helped in the more optimal treatment of patients.

**Main body:**

Many methods have been proposed by researchers for early detection of prostate cancer, but most of the methods used today to detect this type of cancer have been using classical antibodies. Although classical antibodies are able to detect tumor cell markers, but instability, large size, costly and laborious production, and random immobility characteristics, causes many problems. Nanobodies or VHHs, which are derived from camel heavy chain antibodies, have special advantages and have eliminated the disadvantages of classical antibodies which makes them attractive to use in biosensors and cancer diagnostic kits. The research that has been done so far shows that the introduced nanobodies are created for the purpose of targeting, detecting and sensing prostate cancer cells with two main purposes. The first is the efficient identification of prostate cancer and the second is the elimination of cancer cells.

**Conclusion:**

Research shows the use of specific nanobodies against prostate cancer antigens in the design of biosensors and target therapy will be very interesting. In this review article, these nanobodies are introduced and categorized based on their performance.

## Introduction

**Fig. 1:**
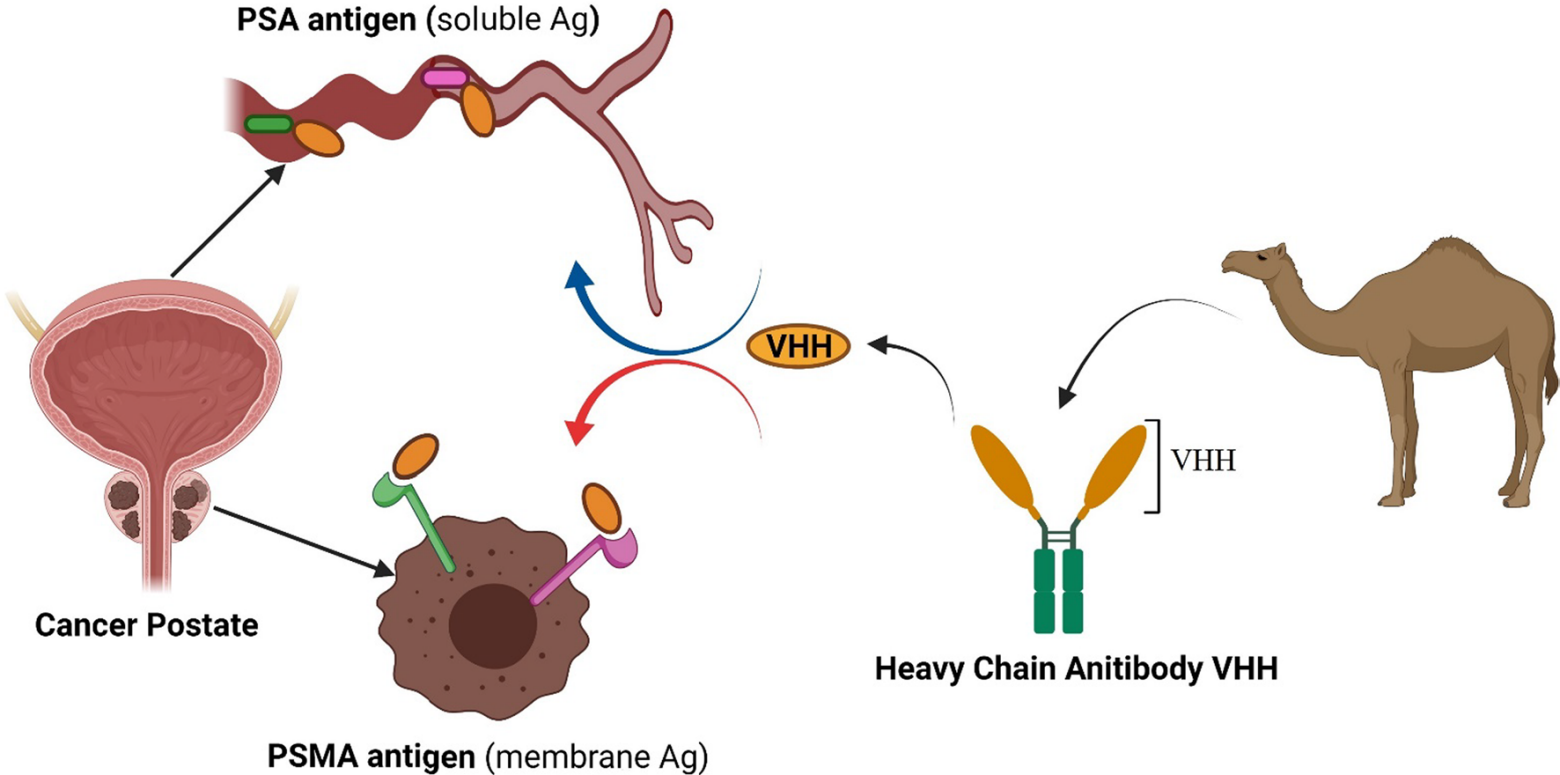
VHH or nanobodies are derived from camel heavy chain antibodies. This nanobodies can detect PSA and PSMA antigen in blood and prostate tissue respectively

Prostate cancer is an important health issue in western countries, and it is the third leading cause of cancer deaths [1, 2]. The prevalence of this malignancy continues to rise in the world [3]. According to reports in Europe, about 450,000 cases of prostate cancer and 107,000 deaths were occurred in 2018 [4]. Due to the lack of effective treatment in the advanced metastatic stage of the disease, early detection of prostate cancer by targeting specific markers is very helpful [2, 3]. Human-specific antibodies, as the most common diagnostic elements, are the obvious choice for cancer targeting and biosensing [2, 5, 6]. Although classical antibodies are able to detect tumor cell markers, but instability, large size, costly and laborious production, and random immobility characteristics, causes many problems [1, 5, 7]. According to studies, more than two thirds of the antibodies produced in the serum of camel species are of the type of heavy chain antibodies (HCAbs) [8]. Nanobodies or VHH which derived from Camelied HCAbs have special advantages such as high stability, small size, and easy and low cost production, that makes them attractive to use in biosensors and cancer diagnostic kits (Fig. 1) [5, 9]. This article reviews nanobodies produced against prostate cancer antigens. These nanobodies are created for the purpose of targeting, detecting and sensing prostate cancer cells. The use of these nanobodies in the design of biosensors will be very interesting. The importance of designing an effective method for detecting cancer cells in their recurrence is even more important because second cancer may occur after the first malignancy [10].

This review article first briefly discusses nanobodies and their properties, then examines the use of nanobodies in prostate cancer in the following three subjects.

1. Nanobodies isolated against prostate-specific antigens.
2. Application of specific nanobodies in diagnosis with imaging methods and biosensor.
3. Application of specific nanobodies in prostate treatment planning.

### Nanobody or VHH

**Fig. 2:**
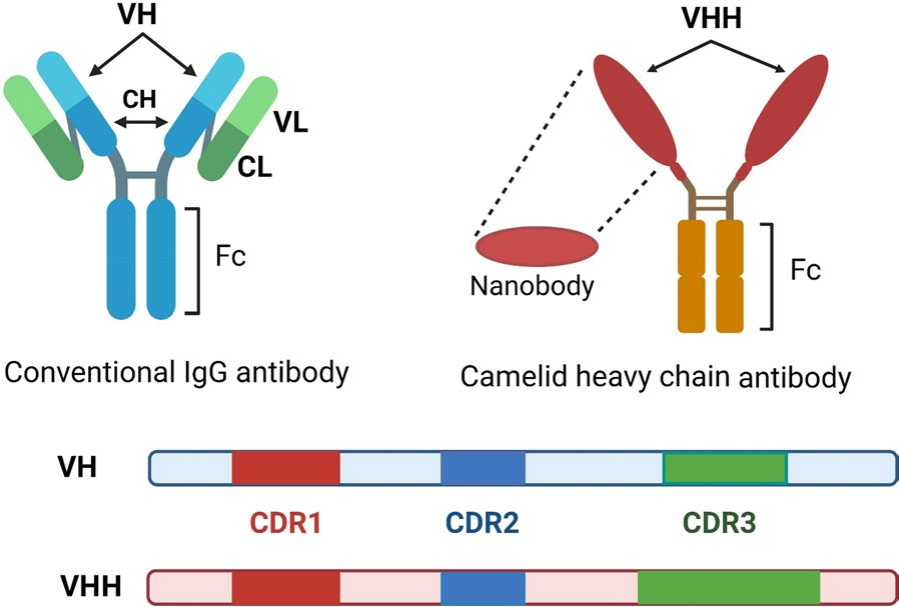
Camelid heavy chain antibodies, unlike conventional antibodies, do not have a light chain and the antigen-detecting part consists only of the variable part of the heavy chain; VHH (Upper). The CDR3 domain of VHH, due to their larger size, allows the detection of cryptic epitopes and haptens, which is not possible in classical antibodies (Lower)

Nanobody or VHH is a group of single-domain antibodies derived from camel heavy chain antibodies (HCAbs) [9]. The antigen binding region of these nanobodies consists of a single chain with three CDRs and four FRs (framework regions); also, the CDR3 domain of these antibodies, due to their larger size, allows the detection of cryptic epitopes and haptens, which is not possible in classical antibodies (Fig. 2) [1, 9, 11]. VHHs, as the smallest antigen-binding fragments (about 15 *K*d), have several properties that make them attractive for use in the diagnosis and treatment of diseases [9]. Manipulation, cloning, and production of VHHs with a high affinity and yields are easily performed on microorganisms (*E. coli* and *S. cerevisiae*), and plant cells due to the lack of a light chain [1, 12, 13]. One of the most important advantages of nanobodies is their resistance to temperature, and alkaline and acidic pH, so nanobody-based methods do not require special storage conditions, and this will reduce costs [9, 14, 15]. Also, the small size of nanobodies leads to lower immunogenicity, better pharmacokinetics, more efficient penetration into tissues and superior targeting of cryptic epitopes [1, 9, 16]. Finally, similarity of nanobodies with the variable part of human antibodies (VH) contributes to their use in clinical applications with the minimal immunogenic reactions [13, 17].

## Searching strategy and methodology

**Fig. 3:**
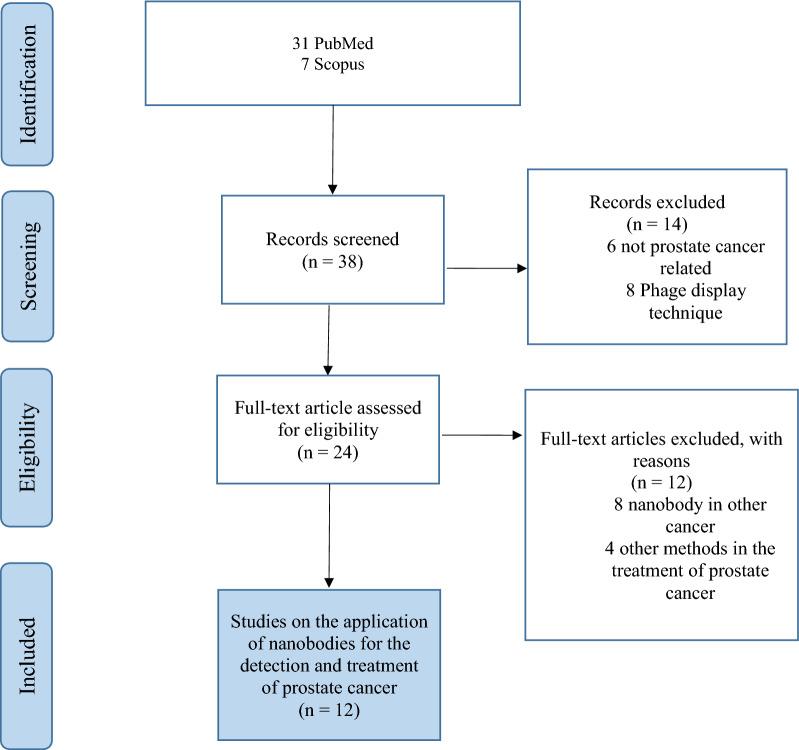
Flow chart of the first literature search according to the PRISMA (Preferred Reporting Items for Systematic Reviews and Meta-Analyses) guidelines

The literature review was performed according to PRISMA instructions [18]. PubMed and Scopus were searched on July 1, 2021 to extract published articles on the use of VHHs in the diagnosis and treatment of prostate cancer. The search key words were prostate cancer, prostate malignancy, PSA, PSMA, nanobody, camelid antibody, single chain antibody, heavy chain anti body, VHH, detection, diagnosis, biosensor, and immunosensor. We used Boolean functions as follows: (Prostate cancer OR Prostate malignancy OR PSMA OR PSA) AND (Nanobody OR Nanobodies OR Camelid antibody OR Single chain antibody OR Heavy chain anti body OR VHH). Two researcher compared the data mining for consistency, and omitted some sources because they did not have the full text or were in the non-English. In this search, 38 studies were obtained. The eligibility of the articles were further examined and irrelevant articles also removed. Finally, 12 articles had inclusion criteria (see Fig. 3).

## Nanobodies against prostate cancer antigens

**Fig. 4:**
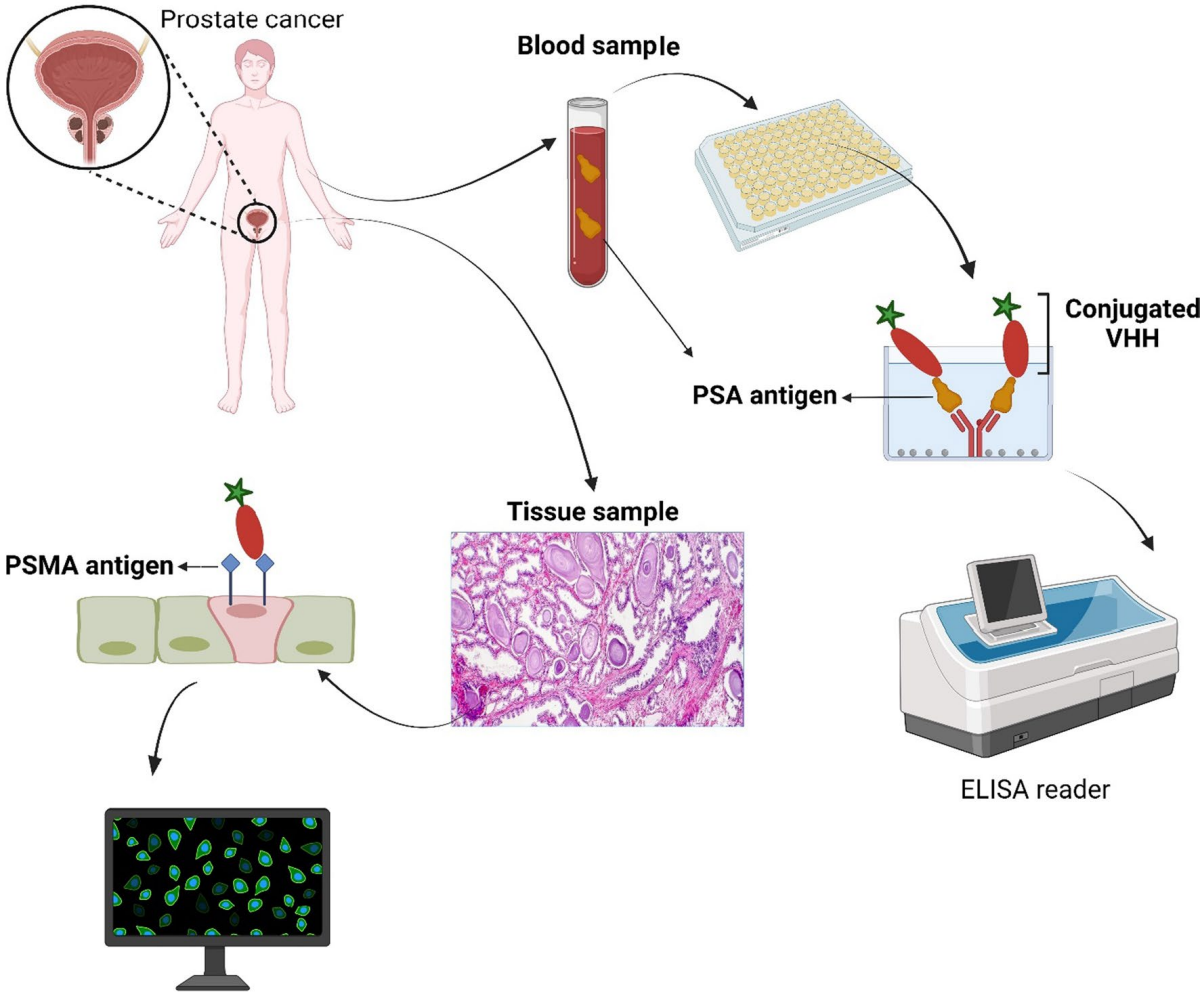
Numerous VHH have been designed and manufactured against prostate cancer antigens, most of which are against PSA (prostate-specific antigen) or PSMA (prostate-specific membrane antigen) antigens. By attaching these nanobodies to reporter molecules, prostate cancer can be detected by ELISA, flow cytometry, immunohistochemistry, etc.

Up to now, several nanobodies have been designed and produced against prostate cancer antigens, most of which have been against PSA (prostate-specific antigen) or PSMA (prostate specific membrane antigen) antigens (Fig. 4). In 2004, Dirk Saerens and colleagues used peripheral blood and lymph node lymphocytes of a dromedary immunized with PSA to produce two gene banks of the VHHs. Various VHHs showed a wide range of kinetic rate constants from 70 pM to 100 nM against free PSA, and *K*d value for best nanobody was 0.16 nM for N7. Some of these VHHs are able to sense structural changes in different PSA isoforms, and this feature can be used to study different stages of prostate cancer. This study suggests that lymph node tissue may be a viable alternative to peripheral blood as a source of cDNA synthesis for a VHH library [19]. Lymph node tissue is easily obtained, and expected to obtain more cDNA than peripheral blood lymphocytes [17]. Although lymph node biopsies may seem complicated, but they should not cause any problems in the veterinary environment. Some of the VHH in this study are obtained from the same B-cell lineage, reflecting the limited primary source of HCAbs. On the other hand, some nanobodies originate from different B-cell lineages, which indicates a strong somatic mutation and strict antigen selection in these animals [19].

In 2012, Mehdi Evazalipour and colleagues produced and characterized a VHH against the external surface domain of the PSMA. This antigen is a membrane glycoprotein, which overexpressed in prostate cancer tissues. In this investigation, dromedary immunization was done by LNCaP cells, recombinant PSMA, and a 28-amino acid peptide, which was consistent with the PSMA external domain. In the following, three camel IgG subtypes including conventional (IgG1 = 160 kDa) and heavy chain (IgG2 and IgG3 = 90 kDa) antibodies were separated by affinity chromatography. IgG3 and IgG1 were separated by protein G column with acetate buffer elution in pH 3.5 and 2.7 respectively; and IgG2 was separated by protein A column with acetate buffer elution in pH 3.5. In this study, high-affinity heavy-chain anti-PSMA antibodies were isolated. The affinity of HCAbs was measured by ELISA and immunocytochemistry, which were 1 × 10^8^ M^−1^ and 6 × 10^7^ M^−1^, for IgG2 and IgG3, respectively [8].

In another study by Zare et al. [1], nanobodies derived from camel antibodies were obtained using phage display technique. Due to the large extracellular domain of PSMA, a part of the extracellular domain of this antigen was selected by bioinformatics tools and this domain, which had suitable antigenic properties, was used to immunize camels. Then, a camel was immunized by recombinant PSMA and LNCaP cell line. VHH was displayed on the surface of M13 phage and this library was used for panning against PSMA. After panning, two VHH-phage clones with high binding affinity were chosen for production of VHH (C3 and C18). The binding affinity of the C3 against rPSMA was 3.5 × 10^7^ M^−1^. This VHH be able to bind to the PSMA antigen on the surface of LnCaP cells. Interestingly, no response was detected when using anti-PSMA VHH on PC3 cells (a PSMA negative cell line). The results of this study showed that nanobodies can be easily displayed on phage particles, and this provides a powerful tool for detecting and isolating VHH with high affinity [1].

## Application of nanobodies in prostate cancer detection by imaging

A radiolabeled nanobody against cancer-specific antigens can be a non-invasive approach to detect patients. In 2013, Evazalipour and colleagues developed a radiolabeled nanobody against PSMA that could detect prostate cancer patients eligible for PSMA-targeted therapies. This VHH were obtained from an immunized dromedary and binding properties were examined by flow cytometry and ELISA test. After examining the ability of nanobodies to bind to PSMA, the VHHs were labeled with at their hexahistidine tail. Specific binding on LNCaP cells was detected for both technetium-99 m labeled PSMA6 and PSMA30 VHHs. Interestingly, the signal was significantly reduced after adding 500-fold molar excess of unlabeled VHH and no binding was detected on PC3 cells. In-vivo tumor targeting was performed in LNCaP xenografted mice by SPECT/micro CT, and two VHH (technetium-99 m labeled PSMA6 and PSMA30) were selected based on ELISA and FACS analysis. The affinity of PSMA6 and PSMA30 were determined 36 nM and 4 nM respectively. It was found that PSMA30 has better targeting and higher degree of internalization both in-vivo as well as in-vitro compared with PSMA6 [4, 8].

In another study by Kristell and Chatalic [20], 111In-radiolabeled anti-PSMA Nanobody was developed for treatment and imaging of prostate cancer. The Nanobody library was created by immunizing the llama with four prostate cancer cell lines (LNCaP, VCaP, MDAPCa-2b, and PC346C), and specific nanobody (JVZ-007) was selected via bio-panning against PSMA antigen. To compare nanobody radiolabeling, the c-terminal his-tag of the nanobody (use in purification and detection) was replaced with a single cysteine, resulting site-specific attachment of the chelates for radiolabeling. In the following, JVZ-007-his and JVZ-007-cys was conjugated to p-SCN-DTPA^1^ and maleimide-DTPA respectively. In vitro cell binding tests to evaluate the targeting of PSMA was performed using autoradiography, flow cytometry, and internalization assays on different prostate cancer cell lines and patient-derived xenografts (PDXs). The results showed that 111In-JVZ-007-his and 111In-JVZ-007-cys transferred to LNCaP cells and attached to PSMA-expressing PDXs. Interestingly, this does not occur in PSMA-negative PDXs. Targeting features of these radiolabeled VHHs were assessed by SPECT/CT imaging and biodistribution methods in mice model bearing PSMA positive (PC-310) and negative (PC-3) tumors. The result demonstrated 111In-JVZ-007-his and 111In-JVZ-007-cys have desirable tumor targeting and rapid blood clearance. The disadvantage of 111In-JVZ-007-his was its high renal uptake, which was efficiently declined by simultaneous injection of lysine and gelofusine. Also, the replacement of his-tag with cysteine reduced the renal uptake of this nanobody, without loss of efficiency [20].

## Application of nanobodies in prostate cancer detection by biosensors

Antibodies are excellent probes in biosensor applications due to their specificity and affinity. Nevertheless, their unstable behavior and large size make several problems. The variable fragment of camelid antibodies (VHH) has special advantages such as high stability, small size, and ease of production, which makes its use in biosensors attractive [5]. These nanobodies are immobilized on the surface of the sensors by covalent bonding, streptavidin–biotin interaction, or metal chelation [5].

In 2005, Huang and colleagues designed a surface plasmon resonance (SPR) biosensor to detect PSA antigen. 24 different nanobodies with specificity for PSA were selected by solid-phase ELISA. Among these, two molecules cAbPSA-N7 and cAbPSA-C23 were selected for further investigation due to their high affinity for PSA (*K*d values of 80 and 70 pM, respectively). Although the equilibrium binding constant of the two nanobodies was almost the same, the kinetic parameters were different, so that the *k*on was 10 times higher (1.9 × 10^6^ M^−1^ S^−1^) and the *k*off was 10 times lower for the cAbPSA-N7. Detection of different low PSA concentrations with these two nanobodies which was placed on a Ni–NTA-biochip through their His-tail, showed that cAbPSA-N7 provides the most sensitive PSA detection signals. The cAbPSA-N7 was covalently immobilized on a gold substrate through a mixed SAM (self-assembled monolayer) of alkanethiols. To optimize the coupling conditions, the prepared nanobody was loaded on coupling buffers with different pH (3.0 to 8.0) on a gold surface coated with a homogeneous 16-MHA (mercapto-1-hexadecanoic acid) monolayer, and the best result was obtained in 10 mM acetate buffer pH 5.5. To determine the optimum composition of the SAM mixture for maximum coupling of cAbPSA-N7 nanobody, different percentages of 16-MHA (100 to 1% v/v) were deposited on gold-coated SPR chips. The findings showed that the highest functional immobilization rate of cAbPSAN7 (1250 RU) was obtained on the 10% (v/v) 16-MHA mixed SAM. This biosensor is able to detect the concentration of PSA up to 10 ng/ml. In the following, detection of limit improved in the sub ng/ml range by using a sandwich method which includes a biotinylated secondary antibody and streptavidin-modified gold nanoparticles [21].

In another study, Saerens et al. [5], show the potential of nanobodies in sensing PSA by SPR technology. In this investigation, BAD-tag, extra Lys residues, and His6-tag were attached to a specific nanobody against the PSA (cAbPSA-N7), for immobilization on the streptavidin chips, SAM chips, and Ni–NTA, respectively. The results showed that these biosensors were able to detect PSA at concentrations below 1 ng/ml in 15 min [5].

In addition, Liu and colleagues developed [6] a sandwich-type biosensor for PSA detection in serum samples based on the two nanobodies.

Following the enrichment of an immune library with 10^8^ individuals, two high-affinity PSA-specific nanobodies (Nb2 and Nb40) were isolated. The small size of Nb40 increases the adsorption level in a reduced graphene oxide nanocomposite with gold nanoparticles (rGO@AuNPs). Special and suitable morphology of providing a wide surface for anchoring and immobilization of many nanobodies. Also, the effective electrical conductivity of rGO accelerated electron transfer on the electrode surface, which is essential for improving the sensitivity of the biosensor. Nb40 as a capture nanobody were attached to rGO@AuNPs/GCE (glassy carbon electrode) surface through the interaction between sulfhydryl/amine groups of gold nanoparticle and nanobody. After PSA capturing, the sandwich system is then performed by the second antibody (Nb2) that were labelled with streptavidin-binding peptide (SBP) tag. This immunosensor had limit of 0.08 ng mL^−1^ and was able to detect PSA concentrations in the range of 0.1 to 100 ng/ml and showed excellent stability and selectivity [6]. Table 1 summarizes the nanobodies in prostate cancer antigen detection by imaging and biosensors.

**Table 1:** The nanobodies studied in this paper with diagnostic application against prostate cancer

Name and type of nanobody	Type of application	Binding affinity (Kd)	References
VHH against PSA	Detection	100 nM–70 pM	Saerens et al. [19]
VHH against external domain of PSMA	Detection	10 nM	Evazalipour et al. [8]
VHH against external domain of PSMA	Detection	35 nM	Zare et al. [1]
A radiolabeled (technetium-99 m) nanobody against PSMA	Imaging	36 nM	Evazalipour et al. [4]
technetium-99 m anti-PSMA nanobody	Imaging	4 nM	Evazalipour et al. [4]
111In-radiolabeled anti-PSMA nanobody	Imaging	N/A	Kristell and Chatalic [20]
Nanobody against PSA (cAbPSA-N7)	Biosensor	70 pM	Huang et al. [21]
Nanobody against PSA (cAbPSA-C23)	Biosensor	80 pM	Huang et al. [21]
Nanobodies against PSA	Biosensor	1 nM	Saerens et al. [5]
Nanobodies against PSA	Biosensor	100 pM–10 nM	Liu et al. [6]

## Nanobodies in prostate cancer target therapy

In a study of anti-PSMA nanobodies in combination with nanobubbles in the diagnostic method of ultrasonography well differentiated prostate cancer cells in comparison with the control group and it was found that this combination is a good method for imaging prostate cancer for diagnostic and therapeutic applications. Nanobubbles are small objects the size of viruses that have many applications in therapeutic delivery. The nanobubbles used in this study were approximately 487.60 ± 33.55 nm in size, which were very small and attached to the anti-PSMA nanobody by the biotin-streptavidin system. After tumor formation of nude mice inoculated with LNCaP and C4-2 cells and the use of MKN45 cells as controls, nanobubbles made for ultrasound imaging were used. In this study, four indicators were considered for the produced nanobubbles and the results showed that the use of these nanobubbles in prostate cancer ultrasonography method is notably different from blank nanobubbles in terms of these four indicators (peak time, peak intensity, arrival time and enhanced duration). This method can be used to identify prostate cancer tissue and target therapy [22].

The results of studies have shown that PSMA is a proper target for the treatment and radionuclides imaging of metastatic prostate cancer. In a study by Nonnekens et.al, two molecules of PSMA I&T (chemical structure) and JVZ-008 (nanobody structure) that had a good binding efficacy to PSMA were selected and attached to an α-emitting radionuclides (213Bi) for greater effect on prostate cancer cells. α-particles have more energy than β particles and can break down the double-stranded DNA of cancer cells, causing deletions, chromosomal perversion and cell death. The results of these researchers showed that the molecules made in this study were able to break down double-stranded DNA (DSBs) in cancer cells in invitro and invivo assay, and are a good target for the treatment and diagnosis of this type of cell [23].

In some cancer cells after administration of chemotherapy, these cells become resistant to medication and make the treatment process difficult for the patients. In a study by Tieu et al., They used biocompatible porous silicon (pSi) nanoparticles that simultaneously carried the doxorubicin and a siRNA against the MRP1 (multidrug resistance-related proteins) gene. In the next step, the nanoparticle was attached to the nanobody against PSMA with a PEG linker to deliver its cargo specifically to prostate cancer cells. The results showed that this intelligent nanoparticle was able to inhibit up to 74% of MRP1 gene expression in prostate cancer cells and inhibit cell resistance to doxorubicin [24].

In a study by Rosenfeld et.al, researchers first isolated several nanobodies against PSMA that had two specific properties. First, they had a higher affinity for PSMA (*K*d: 0.055 nm–6.0 nm) and second, after binding to PSMA, they became internalized, which is an important property for the specific delivery of drugs and fluorescent substances into cancer cells. In the next step, the nanobodies were attached to doxorubicin and tested on the prostate tumor of nude mice. The results showed that the combination of nanobody and doxorubicin 42 times more inhibited tumor growth compared to the control group that received doxorubicin alone. To treat prostate tumor cells, a lower concentration of nanobody-doxorubicin is used in the patient, which results in less damage to the patient's normal cells and a reduction in the side effects of chemotherapy [25].

Recently, anti-PSMA nanobodies have been used to make CAR-T cells and their function on prostate cancer cells has been evaluated. The constructed CAR-T cell, which expresses anti-PSMA nanobodies on its surface, was co-cultured with LNCaP cells (PSMA^+^) and DU‐145 cell (PSMA^−^) as negative control. The results indicated that IL2 cytokine production and CD69 surface expression increased and caused 60% of CAR-T cell proliferation. The results of this study show that the use of nanobodies to make specific CAR-T cells against cancer cells is not only possible but also has promising results in immunotherapy of prostate cancer [26].

## Discussion

This article attempts to discuss the steps taken to use nanobodies in the diagnosis and treatment of prostate cancer. Researchers have tried to overcome the difficulties in diagnosing and treating prostate cancer cells by building different molecules based on nanobodies. Measures such as conjugation of specific nanobodies with radioisotopes and chemotherapy agents. The use of PSMA-specific antigen to produce specific nanobodies against this antigen is very promising because most cancers do not have this specific antigen to target them [27].

Nanobodies have many advantages over classical antibodies and even recombinant antibodies that are discussed in this article [27]. Previous studies on the use of VHHs against tumor-associated cell surface antigens show that nanobodies are more sensitive to antigen detection than conventional antibodies [28]. This ability is likely due to the longer CDR (complementarily determining regions) domains in VHHs, which allow for better interaction with antigens [29]. These ideal molecules also have disadvantages, such as fast renal clearance and inducing kidney toxicity. It is clear that if the goal is to use nanobodies to diagnose prostate cancer and build biosensors, these defects are not problematic and do not impair the diagnosis of cancer. But in the therapeutic application of nanobodies, these disadvantages can be problematic. Various solutions have been proposed by scientists to reduce these obstacles. For example, several methods have been proposed to increase the half-life of nanobodies in the blood and decrease renal clearance. Production of diabodies molecules by conjugating specific nanobodies against the desired antigen and nanobodies with high binding affinity to human albumin has been done by Ablynx Company and now some nanobodies with this format are in different stages of clinical trials. The next solution is to attach the nanobody to the FC of human antibodies or attach the nanobody to the polyethylene glycol (PEG) [27]. The authors of this article believe that the nanobodies obtained against different epitopes of prostate cancer-specific antigens are sufficiently produced. The next step in this area could be the use of antibody engineering to increase the potency and specificity of existing nanobodies. Molecules that are highly efficient and specific to prostate antigens can be obtained by producing diabodies, triabodies, or tetrabodies forms from existing nanobodies that identify different epitopes and increase blood circulation time. Another area of ​​advancement is the engineering of the nanobodies CDRs to replacement of important amino acids in interaction with specific antigens and the construction of a nanobody with strong and specific binding to the target [30]. Also, in a study by Maassa et al., in order to produce a rich library that represents a wide range of nanobodies; more than 50 alpha-HcAb cDNAs were sequenced to assist in the design of PCR primers [17]. As a result, a very complex library was created that included more diverse types of PSA-binding VHH clones. Finally, considering the potency, efficiency and specificity of this type of antibodies, it seems that in the near future, the use of nanobodies in the diagnosis and treatment of diseases such as cancer will be more widespread.

## Conclusion

Camel heavy chain antibodies (VHH) or nanobodies have special advantages such as high stability, small size, and easy and low cost production, which makes them attractive to use in biosensors and cancer diagnostic kits. These nanobodies produced against prostate cancer antigens are created for the purpose of targeting, detecting and sensing prostate cancer cells. The use of these antibodies in the design of biosensors will be very interesting.

## Abbreviations

PSMA: Prostate-specific membrane antigen
PSA: Prostate-specific antigen

## Footnotes

1 2-(4-isothiocyanatobenzyl)-diethylenetriamine pentaacetic acid.
